# Longitudinal Effects of Bilateral Subthalamic Nucleus Deep Brain Stimulation Versus Best Medical Therapy on Static and Dynamic Balance and Gait in Advanced Parkinson’s Disease: A 36-Month Comparative Study

**DOI:** 10.3390/biomedicines13112794

**Published:** 2025-11-17

**Authors:** Stanisław Szlufik, Maria Kłoda, Karolina Jaros, Iwona Potrzebowska, Łukasz Milanowski, Monika Figura, Tomasz Mandat, Dariusz Koziorowski

**Affiliations:** 1 Department of Neurology, Faculty of Health Sciences, Medical University of Warsaw, 02-091 Warsaw, Poland; 2 Department of Neurosurgery, Maria Sklodowska-Curie National Research Institute of Oncology, 02-781 Warsaw, Poland; 3 Department of Physiotherapy Fundamentals, Faculty of Dental Medicine, Medical University of Warsaw, 02-091 Warsaw, Poland

**Keywords:** Parkinson’s disease, deep brain stimulation, static balance, dynamic balance, gait disturbances, posturography, subthalamic nucleus, neuromodulation

## Abstract

**Objective**: To evaluate the long-term impact of bilateral subthalamic nucleus deep brain stimulation (STN-DBS) versus best medical therapy (BMT) on static and dynamic balance as well as gait disturbances in patients with advanced Parkinson’s disease (PD). **Methods**: In this prospective study, 50 patients with advanced PD were randomly assigned to receive either bilateral STN-DBS (n = 28) or BMT (n = 22). Comprehensive evaluations were performed at baseline and during four consecutive visits over 36 months. Static balance was assessed using posturographic measurements (COP velocity, perimeter, ellipse area), and dynamic balance and gait were evaluated using tandem gait tasks and pivoting maneuvers. Statistical analyses included repeated measures ANOVA-Friedman and Dunn-Bonferroni post hoc tests. **Results**: DBS-treated patients demonstrated stable dynamic balance and gait performance over 36 months with no significant decline in tandem gait and pivot tests. Conversely, the BMT group showed a significant deterioration in dynamic balance (Walking Tandem Test; χ^2^ = 10.63, *p* = 0.014) and gait function, particularly in the medication OFF state. Static balance in the DBS group worsened notably under sensory-deprived conditions (eyes closed, OFF state; χ^2^ = 10.13, *p* = 0.017), whereas BMT maintained static balance stability without significant changes. **Conclusions**: STN-DBS effectively preserves dynamic balance and gait functions in patients with advanced PD over 36 months, but exhibits limited efficacy in maintaining static balance under sensory-deprived conditions. These findings highlight the need for individualized therapeutic approaches that emphasize combined neuromodulation and multidisciplinary rehabilitation strategies.

## 1. Introduction

Parkinson’s disease (PD) is among the most prevalent neurodegenerative disorders, and current projections estimate that by 2050, it will affect over 25 million individuals globally, thereby posing a significant and escalating public health challenge [1]. PD is a chronic, progressive disorder characterized by cardinal motor symptoms, including resting tremor, rigidity, and bradykinesia, along with a spectrum of non-motor manifestations, such as sleep disorders, hyposmia, constipation, pain, and neuropsychiatric dysfunctions [2,3]. Balance disturbances and gait impairment are among the most debilitating hallmarks of PD and substantially contribute to morbidity and reduced functional independence. Postural instability typically emerges at a median of 4.7 years post-diagnosis and affects two-thirds of the patients by 10 years following diagnosis [4]. These impairments significantly increase the risk of falls and fall-related injuries. Epidemiological data indicate that 35–90% of patients with PD experience at least one fall, with an average incidence of 60.5%, and approximately 39% are classified as recurrent fallers [5]. Falls in this population are associated with a markedly elevated risk of fractures compared with individuals with other chronic medical conditions [6]. Motor complications are a major determinant of the deterioration of health-related quality of life in patients with PD, and their impact tends to intensify with disease progression [7,8,9]. Although dopaminergic medications remain the cornerstone of symptomatic management, their efficacy diminishes in the advanced stages of the disease. Motor complications are a principal contributor to long-term levodopa therapy, which although initially effective, is associated with the emergence of motor fluctuations and dyskinesias in approximately 40–50% of patients after 4–6 years of treatment [10].

As patients progress to the advanced stage of PD, oral pharmacotherapy frequently becomes inadequate to maintain satisfactory control over motor symptoms and associated complications [11]. Deep brain stimulation (DBS), particularly targeting the subthalamic nucleus (STN), has emerged as a well-established and effective therapeutic option for patients with advanced PD [12] and is typically characterized by the emergence of motor complications such as fluctuations and dyskinesias [13]. STN-DBS has demonstrated robust and sustained efficacy in ameliorating the cardinal motor symptoms of PD, with clinical benefits reported to persist for more than 15 years in many cases [14]. However, its impact on gait disturbances and postural instability remains controversial. While some patients experience significant improvements in axial symptoms following DBS [15], others may develop new gait abnormalities or experience worsening of balance impairments over time [16]. These divergent outcomes underscore the complexity of axial symptomatology in PD and highlight the need for further investigation, particularly in the context of long-term follow-up.

The objective of this study was to evaluate the long-term effects of bilateral (STN-DBS) on gait disturbances and postural instability in patients with PD. To accomplish this, a comparative analysis was conducted between a cohort of PD patients receiving Best Medical Therapy (BMT) and a cohort who underwent bilateral STN-DBS implantation.

## 2. Materials and Methods

The participants in this study were individuals diagnosed with idiopathic PD, all of whom met the MDS Clinical Diagnostic Criteria for Parkinson’s Disease [2]. Eligibility for inclusion required patients also to meet the criteria set by the Core Assessment Program for Surgical Interventional Therapies in Parkinson’s Disease (CAPSIT-PD) [17], qualifying them for bilateral STN-DBS. All participants demonstrated a sustained response to levodopa accompanied by motor complications, including disabling motor fluctuations with extended and occasionally unpredictable OFF periods, during which patients spent 25% of their waking hours in the OFF state and ON state dyskinesias.

Participants were divided into two groups:

(1) The Best Medical Therapy (BMT) cohort comprised 22 patients who were randomly assigned to not undergo STN-DBS surgery, despite meeting the eligibility criteria for the procedure. This cohort, with an average age of 64.3 years, was treated solely with medication during the study. The aim of this group was to assess the usual progression of static and dynamic balance disorders in patients with PD who were managed only with pharmacotherapy, without the use of device-assisted treatments.
(2) The DBS surgery cohort included 28 patients with an average age of 58.3 years. The patient demographics are presented in Table 1. Allocation to the study groups (DBS vs. BMT) was conducted randomly at a 1:1 ratio, considering the patients’ age and motor status during the ON phase. Upon completion of the study procedures, all the participants in the BMT group were offered the opportunity to undergo surgery. Comprehensive demographic data of the participants are presented in Table 1.

Both groups were assessed over four visits, with the initial visit to the DBS group conducted preoperatively. The time interval between visits was scheduled at 12-month intervals and was comparable in both groups. Evaluations were carried out by a clinician specializing in movement disorders, using the Unified Parkinson’s Disease Rating Scale part III (UPDRS III). Patients underwent gait and posturographic assessments, as well as balance tests, administered by physiotherapists experienced with movement disorders. Gait analysis was performed on a moving treadmill at speeds of 1.5 km/h and 3.5 km/h. Biofeedback and posturographic assessments were conducted on a stabilometric platform, with evaluations performed under two conditions: eyes open and closed. Balance tests included the Timed “Up and Go” test (TUG), Tandem Stance test (TST), 180° Tandem Pivot test (TPT), and Tandem Walking test (TWT). Measurements for the BMT group and the preoperative visit of the DBS group were taken during the ON medication state, following the administration of their standard drug dose, and during the OFF medication state, after a minimum of 12 h without levodopa and 24 h without any other antiparkinsonian medication. The DBS group underwent four assessments in total: twice during the ON medication stage with both stimulators activated (DBS ON) and deactivated (DBS OFF), and twice during the OFF medication stage with both stimulators activated (DBS ON) and deactivated (DBS OFF). Evaluations in the DBS OFF stage were conducted 30 min after stimulation deactivation. The STN-DBS surgery was performed without complications throughout the study period. During the entire follow-up period, stimulation parameters were individually optimized and frequently adjusted over the 36-month follow-up period to maximize clinical benefits and minimize side effects. Therefore, a uniform set of stimulation values cannot be reported. Nevertheless, most patients were stimulated within the commonly recommended therapeutic ranges, typically using frequencies of approximately 130 Hz, with amplitudes and pulse widths tailored individually.

This study was conducted in accordance with the Declaration of Helsinki and based on the recommendations of the Bioethics Committee of Warsaw Medical University. Written informed consent was obtained from all the participants. The study protocol was approved by the Bioethics Committee of Warsaw Medical University (approval code: KB/160/2014).

Data analysis was conducted using the STATISTICA version 13.5 software. The Shapiro–Wilk test was used to evaluate the normality of the distribution. Continuous variables were expressed as means and odds ratios, while categorical variables were shown as frequencies with percentages. Independent t-tests were used to compare parametric data. The ANOVA-Friedman test was used to assess repeated measures, and the Dunn-Bonferroni test was used for post hoc analysis.

## 3. Results

The comprehensive statistical analysis conducted in this study revealed significant differences in posturographic parameters and balance tests across the four follow-up visits in patients with PD treated with either DBS or Best Medical Therapy (BMT).

**Figure 1:**
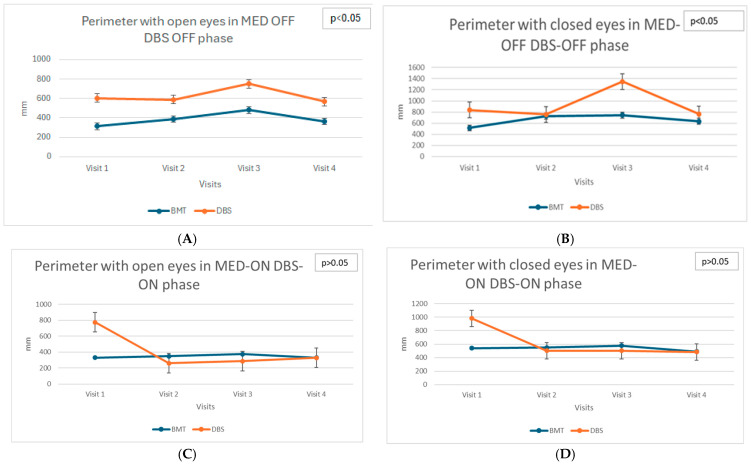

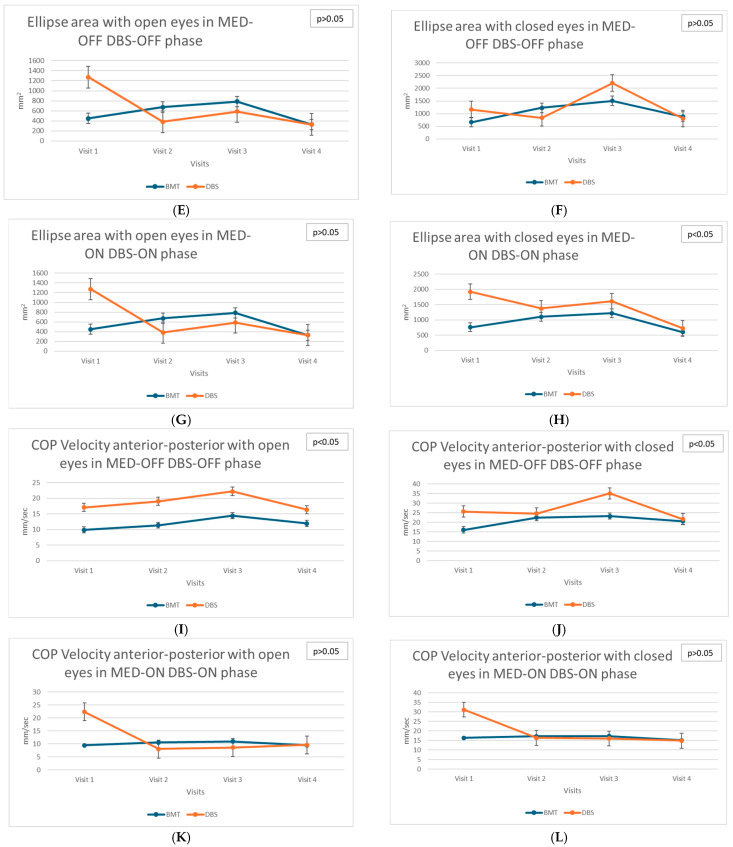
(**A**)—Longitudinal assessment: Perimeter with open eyes in MED-OFF DBS-OFF phase. (**B**)—Longitudinal assessment: Perimeter with closed eyes in MED-OFF DBS-OFF phase. (**C**)—Longitudinal assessment: Perimeter with open eyes in MED-ON DBS-ON phase. (**D**)—Longitudinal assessment: Perimeter with closed eyes in MED-ON DBS-ON phase. (**E**)—Longitudinal assessment: Ellipse area with open eyes in MED-OFF DBS-OFF phase. (**F**)—Longitudinal assessment: Ellipse area with closed eyes in MED-OFF DBS-OFF phase. (**G**)—Longitudinal assessment: Ellipse area with open eyes in MED-ON DBS-ON phase. (**H**)—Longitudinal assessment: Ellipse area with closed eyes in MED-ON DBS-ON phase. (**I**)—Longitudinal assessment: COP Velocity anterior–posterior with open eyes in MED-OFF DBS-OFF phase. (**J**)—Longitudinal assessment: COP Velocity anterior–posterior with closed eyes in MED-OFF DBS-OFF phase. (**K**)—Longitudinal assessment: COP Velocity anterior–posterior with open eyes in MED-ON DBS-ON phase. (**L**)—Longitudinal assessment: COP Velocity anterior–posterior with closed eyes in MED-ON DBS-ON phase.

Static balance parameters assessed using stabilometric measures, such as Perimeter of the center of pressure (COP), Ellipse Area (PoleElipsy COP), and mean velocities of COP displacement (both anterior–posterior and lateral), showed differentiated trends in the DBS and BMT groups across the different phases of medication (MED-ON and MED-OFF) and stimulation status (DBS-ON, DBS-OFF) what indicates the Total ON (MED-ON DBS-ON) and Total OFF (MED-OFF DBS-OFF) states (see Figure 1A−L).

In the DBS group, significant deterioration of static balance, particularly evident in the increased anterior–posterior COP velocity in the TOTAL OFF condition with eyes closed (χ^2^ = 10.13; *p* = 0.017), was observed. This finding highlights the progressive decrease in postural stability without visual input. The increasing velocity of COP movement indicates worsening postural control and increased instability during successive visits after DBS surgery. These changes suggest the limited efficacy of DBS in improving static balance control, particularly when patients rely exclusively on somatosensory and vestibular systems for balance maintenance.

In contrast, the BMT group demonstrated relative stability in static balance parameters as no significant changes were detected across visits. This indicates that standard pharmacological therapy might sufficiently maintain static postural control in patients with PD, although no clear improvement was observed.

**Figure 2:**
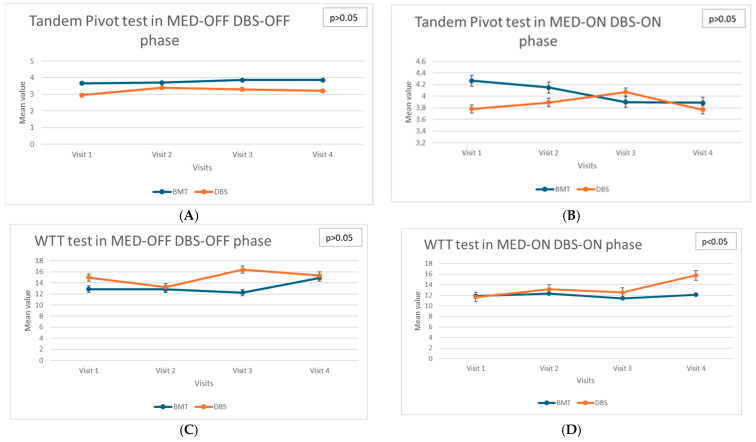
(**A**)—Longitudinal assessment: Tandem Pivot test in MED-OFF DBS-OFF phase. (**B**)—Longitudinal assessment: Tandem Pivot test in MED-ON DBS-ON phase. (**C**)—Longitudinal assessment: WTT in MED-OFF DBS-OFF phase. (**D**)—Longitudinal assessment: WTT in MED-ON DBS-ON phase.

Dynamic balance assessments, including the Walking Tandem Test (WTT) and Tandem Pivot tests, provided insights into the complex relationship between treatment modalities and dynamic postural control (see Figure 2). The Tandem Stance Test (TST) and Timed Up and Go (TUG) were performed but did not yield significant longitudinal differences and thus were not included in the figures.

The DBS group exhibited stability across follow-up visits in these dynamic tests, indicating a preserved dynamic balance control. Specifically, performance on the tandem pivot tasks remained consistent throughout the study period, with no statistically significant differences detected (χ^2^ = 2.91; *p* = 0.406). This result suggests a beneficial effect of DBS in maintaining dynamic stability and preventing the functional deterioration of balance during complex motor tasks. In contrast, significant deterioration in the Walking Tandem Test (WTT) performance was observed in the BMT group in the TOTAL OFF condition (χ^2^ = 10.63; *p* = 0.014). This progressive impairment highlights the limited long-term effectiveness of pharmacological therapy in preserving the dynamic balance during challenging locomotor tasks. Patients receiving BMT experienced increased difficulty in maintaining tandem gait, reflecting the advancing severity of parkinsonian symptoms affecting postural stability and motor coordination.

Additionally, dynamic postural control, measured using the Ellipse Area parameter under eyes-closed conditions in the TOTAL ON state, significantly deteriorated in the BMT group (χ^2^ = 8.29, *p* = 0.040). This suggests that, despite pharmacological treatment aimed at optimizing dopaminergic stimulation, these patients exhibited progressive difficulty in maintaining dynamic postural stability, which may reflect advancing disease pathology and decreasing responsiveness to dopaminergic medications over time.

In contrast, the DBS group showed no significant changes in the same parameter, indicating that DBS effectively mitigates the progression of dynamic balance impairments under optimal medication and stimulation conditions.

Gait disturbances, assessed through tandem gait tasks and pivoting maneuvers, provided critical information regarding motor control and ambulatory stability. In the DBS group, gait parameters were stable throughout the four follow-up visits, reflecting the effective control of motor symptoms affecting gait. The consistent performance on these motor tasks underscores the protective role of DBS in mitigating gait disturbances associated with PD. Conversely, the BMT group showed a significant deterioration in the Walking Tandem Test, highlighting progressive impairments in gait and coordination despite pharmacological treatment.

The findings of this comprehensive longitudinal study indicate the differential effects of DBS and BMT on various dimensions of balance and gait control in patients with advanced PD. Although DBS appears to exert protective effects on dynamic balance and gait control, maintaining functional stability over extended periods, it simultaneously shows limited efficacy or potential deterioration in static balance control, particularly under sensory-deprived conditions. Conversely, standard pharmacological therapy while maintaining static balance relatively effectively is insufficient to halt the progressive deterioration of dynamic balance and gait control. These findings emphasize the complex interplay between the treatment modality, disease progression, and the specific dimensions of postural and motor control affected by PD.

## 4. Discussion

The present study comprehensively investigates the long-term effects of bilateral STN-DBS compared to best medical therapy (BMT) on static and dynamic balance, as well as gait disturbances in patients with advanced PD. Our findings clearly highlight the differential outcomes in balance and gait control, underscoring the complexity of therapeutic interventions for advanced-stage PD. Static balance control, evaluated through detailed posturographic analysis such as center of pressure (COP) velocity, perimeter, and ellipse area, showed significant deterioration in DBS-treated patients over the four visits, especially under conditions of sensory deprivation (eyes closed, medication OFF state). This notable increase in anterior–posterior COP velocity illustrates a gradual decline in static postural stability, indicating that STN-DBS may not sufficiently address axial symptoms such as postural instability in advanced PD stages. A recent systematic review by Janssen et al. [16] consistently highlighted that a subset of patients who underwent STN-DBS experienced worsening balance post-surgery. Such deterioration may result from stimulation parameters, suboptimal electrode placement, or individual patient characteristics including age and disease duration prior to surgery. Our findings support these observations, emphasizing the need for individualized parameter optimization and electrode targeting to mitigate the axial symptoms.

An additional dimension to consider is the role of sensory re-weighting under visual deprivation: when vision is unavailable, the postural control system tends to upregulate reliance on somatosensory and vestibular inputs, which may magnify subtle deficits in these modalities. For example, studies in healthy and pathological populations show that closing the eyes often leads to increased postural sway or altered sway dynamics, particularly when proprioceptive or vestibular feedback is impaired [18]. In Parkinson’s disease, there is evidence that STN-DBS may improve balance performance in sensory input–inconsistent or deprived conditions, presumably by restoring better integration of remaining afferent signals [19]. Thus, our findings of different behavior in “eyes closed” vs. “eyes open” conditions could reflect not just baseline imbalance, but also differential sensory weighting strategies under DBS and BMT. This nuance may help explain why some postural parameters diverged more in the deprived state than when visual cues were available.

Conversely, the BMT group demonstrated a stable pattern of static balance parameters throughout the study period. Although no significant improvement was recorded, pharmacological management appeared to prevent further deterioration, indicating the effectiveness of dopamine replacement therapies (DRTs) in maintaining static postural control. The literature consistently recognizes the limitations of dopaminergic therapies in addressing axial symptoms, particularly in advanced PD [20]. In dynamic balance tests such as the Walking Tandem Test (WTT) and Tandem Pivot, the DBS group exhibited remarkable stability over the follow-up period, suggesting a protective role of DBS in dynamic postural control. This sustained ability to maintain balance during complex locomotor tasks aligns well with evidence provided by Jost et al. [21], indicating significant functional gains in quality of life and mobility post-DBS surgery. Our results further validate the clinical utility of DBS in enhancing dynamic motor performance, likely through its modulatory effects on the basal ganglia-thalamocortical pathways involved in gait initiation and execution. Recent neuroimaging studies have suggested that DBS might normalize pathological oscillatory activity within these motor circuits, thus improving motor coordination and reducing freezing of gait episodes [22]. This mechanism could explain the preservation of the dynamic balance abilities observed in patients treated with DBS. Additionally, previous longitudinal studies support the long-term efficacy of STN-DBS in stabilizing axial symptoms, particularly those related to gait and postural adjustments during movement tasks, which significantly contribute to improved functional independence and overall patient autonomy [23]. Thus, the present study reinforces the critical role of DBS as a therapeutic intervention not only in alleviating primary motor symptoms, but also in maintaining complex dynamic balance tasks crucial for daily functioning.

In contrast, the BMT group displayed significant deterioration in WTT performance in the medication OFF state, highlighting the inadequacy of long-term pharmacological treatment in preserving dynamic balance under challenging conditions. These findings complement previous literature emphasizing that medication alone often fails to halt the progression of dynamic balance and gait impairments, especially in complex or demanding tasks that require precise motor control [11]. Such deterioration in dynamic balance likely results from progressive neurodegeneration affecting non-dopaminergic systems, including cholinergic and noradrenergic pathways, which play critical roles in gait and postural control, especially during challenging or attention-demanding tasks [24]. Moreover, the decline in WTT performance observed aligns with clinical observations, indicating that long-term dopamine replacement therapy can lead to diminished efficacy, motor fluctuations, and dyskinesias, ultimately impacting the ability of patients to execute precise and coordinated movements [25]. Importantly, evidence suggests that advanced-stage PD patients often require adjunct therapies to adequately manage axial symptoms, as pharmacological therapies primarily target dopamine-responsive motor features rather than axial instability and gait disturbances [26]. The progressive deterioration observed in our BMT cohort underscores the necessity of integrated management approaches, potentially combining pharmacotherapy with physiotherapy, cognitive training, and device-assisted therapies, to comprehensively address dynamic balance deficits and enhance overall functional capacity in patients with PD [27].

The different therapeutic outcomes observed in this study underscore the importance of personalized management strategies. Considering the strengths and limitations of DBS and BMT, clinicians should carefully evaluate individual patient profiles, including baseline motor and axial symptoms, cognitive function, and age to optimize therapeutic choices. A personalized approach, guided by detailed clinical assessments, could maximize therapeutic benefits and minimize the risk of adverse outcomes associated with treatment selection and implementation [13]. Indeed, recent clinical guidelines emphasize the necessity for individualized, patient-centered decision-making, especially when considering invasive treatments such as DBS [12].

Future research should address critical gaps in the understanding of the precise neural mechanisms by which DBS influences dynamic versus static balance. Advanced imaging techniques, such as functional magnetic resonance imaging (fMRI), can elucidate how DBS modulates the cortical and subcortical networks implicated in balance and gait control. Identification of neural biomarkers predictive of therapeutic responses to DBS would enhance clinical decision-making and improve patient outcomes by allowing clinicians to better select suitable candidates for DBS therapy.

Specifically, fMRI studies have the potential to reveal the modulatory effects of DBS on cortico-basal ganglia-thalamocortical loops, which are crucial in motor planning and execution. Recent research indicates that DBS might normalize aberrant oscillatory activity and hypersynchronization within these networks, thus restoring more physiological patterns of neuronal communication and motor coordination [28]. Such normalization of neural activity could potentially explain why dynamic balance tasks that demand higher-level cognitive engagement and rapid motor adjustments may benefit more significantly from DBS than static tasks, which may depend more heavily on different neural mechanisms such as proprioceptive and vestibular integration [29].

Electrophysiological techniques, such as local field potentials (LFPs) recorded from implanted DBS electrodes, also offer significant promise in unraveling the mechanisms underlying DBS’s effects on balance control. Analysis of LFP patterns has revealed specific frequency band alterations, including beta-band suppression and gamma-band enhancement, which are correlated with motor symptom improvement and gait function restoration [30]. Monitoring such electrophysiological biomarkers could potentially enable clinicians to optimize DBS settings in real-time, further enhancing therapeutic effectiveness.

Understanding the role of cerebellar connectivity in DBS-mediated balance improvement is a critical avenue for future research. The cerebellum plays a vital role in balance and coordination by integrating proprioceptive, vestibular, and visual inputs to maintain postural stability and smooth motor execution [31]. Recent imaging studies indicate altered functional connectivity between the cerebellum and basal ganglia in PD, suggesting that DBS might partially restore disrupted cerebellar-thalamic-basal ganglia circuits, thus improving dynamic balance control [32]. Clarifying these cerebellar interactions could lead to novel therapeutic targets and refine DBS protocols tailored specifically to enhance balance and gait outcomes.

In addition to imaging and electrophysiological approaches, computational modeling offers another valuable tool for elucidating the neural mechanisms of DBS. Detailed computational models incorporating patient-specific anatomy and connectivity patterns could simulate how various DBS parameters affect the neural circuitry involved in balance control [33]. Such models could predict individualized therapeutic responses, guide clinical decision-making, and provide mechanistic insights that could be tested experimentally, further enhancing personalized treatment strategies.

Ultimately, combining advanced imaging, electrophysiological, and computational modeling approaches could deepen our understanding of how DBS affects dynamic and static balance. Such multidisciplinary integration promises to accelerate the development of more effective DBS strategies tailored to individual patient needs, thereby significantly improving the quality of life of patients with PD.

## 5. Conclusions

In conclusion, our comprehensive analysis clearly delineates the therapeutic profiles of DBS and BMT in advanced PD, highlighting DBS’s robust protective effect on dynamic balance and gait functions, alongside potential limitations in static balance control. These insights significantly contribute to clinical strategies, emphasizing the need for individualized treatment paradigms to maximize therapeutic outcomes. Continuous advancements in neuromodulation techniques and comprehensive multidisciplinary rehabilitation protocols promise to enhance DBS efficacy further. Future studies should focus on optimizing patient selection criteria, refining stimulation parameters, and integrating adjunctive therapies to effectively manage static balance limitations. Such approaches will ensure improved quality of life, sustained functional independence, and better long-term outcomes in patients with advanced PD.

## Figures and Tables

**Table 1:** Detailed demographic and clinical characteristics of patients.

Group	Number of Patients	Gender	Mean Age (Years)	Mean Disease Duration (Years)	Mean LEDD (mg/day)	Mean UPDRS III ON	Mean UPDRS III OFF
BMT	22	8 male, 16 female	64.29	8.72	1095.62	10.25	32.33
DBS	28	16 male, 10 female	58.31	9.67	1421.73	10.12	38.38

## Data Availability

The original contributions presented in this study are included in the article. Further inquiries can be directed to the corresponding author.
